# A randomized clinical trial testing a health literacy intervention to reduce disparities in access to care among Justice-Impacted Adults (JIA)

**DOI:** 10.1186/s40352-024-00284-7

**Published:** 2024-07-31

**Authors:** Victoria D. Ojeda, Arthur Groneman, Sarah Hiller-Venegas, Melissa Moreno, Briana Schuler, Jerrica Barksdale, Emily Berliant, Natalie Romero, Todd M. Edwards, Zephon Lister, Todd Gilmer, Tommi Gaines, Angela Bazzi

**Affiliations:** Herbert Wertheim School of Public Health, University of California San Diego, 9500 Gilman Drive, La Jolla, CA 92093-0725 USA

**Keywords:** Probation, Parole, Court-diverted, Reentrant, Formerly incarcerated, Health literacy, Access to healthcare, Healthcare utilization, Health coaching

## Abstract

**Background:**

Low health literacy is costly and observed among justice-impacted adults (JIA), a group that often faces numerous barriers in accessing healthcare and a disproportionate burden of illness. Health literacy interventions for JIA are critically needed to improve healthcare access and related outcomes.

**Methods:**

This manuscript describes the protocol for a longitudinal mixed-methods randomized clinical trial that assesses the effectiveness of a coach-guided health literacy intervention on JIA’s healthcare access. The intervention was previously piloted with justice impacted adults. We will recruit 300 JIA ages 18 + in San Diego, California. Participants will be randomized 1:1 to the Treatment Group (i.e., coach-guided intervention providing 12 sessions of individualized health coaching and service navigation over 6 months) or the Control Group (i.e., self-study of the health coaching program, and brief service navigation support). We will quantitatively assess JIA’s healthcare access defined as: use of healthcare, health insurance status, and regular source of care at 6-months as the primary outcomes. Participants will also be surveyed at 12-months. Statistical analyses will incorporate the intent-to-treat (ITT) principle and we will estimate mixed-effects logistic regression for the primary outcomes. We will also conduct qualitative interviews at 6 and 12-months with 40 purposively sampled participants, stratified by study arm, who reported healthcare access barriers at baseline. Interviews will explore participants’ satisfaction with the intervention, healthcare attitudes, self-efficacy for and barriers to healthcare access over time, perceived contribution of the intervention to health and well-being, and diffusion of intervention-related information within participants’ social networks. We will conduct deductive thematic analyses of qualitative data.

**Discussion:**

Low health literacy among JIA is a foundational challenge requiring tailored intervention strategies. Findings from this trial may inform policies and the structure of service delivery models to build health literacy among JIA in institutional and community settings throughout the United States and elsewhere.

**Trial registration:**

This study is registered with the United States’ ClinicalTrials.gov registry under protocol # 161,903.

## Background

The health of justice-impacted adults (JIA; e.g., persons exiting prisons or jails, under community supervision, or in contact with legal systems) can be adversely impacted by multiple factors including low “*personal health literacy*” which refers to the skills that support an individual’s ability to procure, understand, and use resources that support their health and that of other persons (U.S. Department of Health and Human Services, 2023). The need for personal health literacy is high as the adoption of patient-centered care has increased individuals’ responsibilities as healthcare consumers. Patients are expected to draw on visual, quantitative and digital literacy skills in order to use health services properly, communicate effectively with providers, assess risks/benefits of healthcare, and appropriately locate and evaluate information (Kindig et al., 2004; National Institutes of Health Network of the National Library of Medicine, 2020).

While data evaluation the health literacy of JIA are scarce, a small body of qualitative and quantitative studies concur that JIA often face challenges in having the skills (e.g., literacy, numeracy, digital, self-advocacy) or they may encounter barriers to accessing resources (e.g., health information; technology, social support, logistical barriers to healthcare service use) that support health literacy (Donelle & Hall, 2014; Hadden et al., 2018; Mehay et al., 2021; Ramaswamy & Kelly, 2015). Data such as these underscore the need to address multiple intersecting individual and structural conditions in order to support health literacy among JIA.

Addressing health literacy among JIA is important given the higher burden of illness among this sub-group as compared to the general population (Hawks et al., 2020). For example, significant proportions of JIA are impacted by chronic conditions that require regular monitoring and care such as chronic pain (47%), hypertension (34%), asthma (27%), Hepatitis C (24%), arthritis (22%), HIV (14%), and diabetes (13%) (Beckwith et al., 2010; Wang et al., 2012). JIA’s ability to navigate patient-centered care may be complicated by low educational attainment or underdeveloped basic skills (e.g., reading, numeric literacy); moreover, restrictions on self-care in carceral settings may impede development of these skills (Kindig et al., 2004; National Institutes of Health Network of the National Library of Medicine, 2020; Puglisi et al., 2017; Walsh-Felz et al., 2019; Wang et al., 2019). Extant research also finds that low personal health literacy is also associated with reduced use of preventive healthcare, increased use of emergency care, and higher mortality (Berkman et al., 2011; Kindig et al., 2004). Thus, addressing low personal health literacy is a national priority, and strategies tailored to justice impacted adults’ needs are required (U.S. Department of Health and Human Services, 2023).

Two promising strategies that may support JIA and their health literacy include Health Coaching and Service Navigation. Health Coaching is a patient-centered strategy that seeks to build health literacy (Logan et al., 2015) and can be delivered by clinicians or health or peer educators (Ghorob et al., 2013). This approach has been used successfully to help patients manage chronic conditions and improve medication adherence. Health Coaching is associated with improved shared decision-making, information sharing, behavioral changes (Ghorob et al., 2013) and reductions in inpatient and total health care costs (Jonk et al., 2015). Service Navigation may be paired with Health Coaching with the goal of overcoming barriers to services that support well-being (Bernie et al., 2021; Carter et al., 2018; Fox et al., 2014). For example, an estimated 26% of JIA are uninsured (Hawks et al., 2020) and a lack of insurance or a regular source of care can contribute to delaying or forgoing care (Agency for Healthcare Research and Quality, 2018; Hadden et al., 2018). JIA are more likely to utilize emergency care or be hospitalized than the general population (Hawks et al., 2020). JIA also encounter persistent barriers to accessing related services to address the social determinants of health (e.g., employment, housing, nutrition support) and may be challenged when navigating complex systems of care in the community (KUSI Meade & Mellgren, 2011; News, 2020; Patel et al., 2014). Both Health Coaching and Service Navigation can help patients develop or improve executive functioning skills (e.g., goal setting, time management, resource procurement) that are transferrable across diverse settings (e.g., work, school) (Ojeda et al., 2021). Both approaches also provide social support and mentorship and can help individuals build resilience in the face of structural and social challenges (Teggart et al., 2023).

### Pilot study

We first conducted a feasibility study between 2016–2020 in San Diego, California. We piloted a health literacy intervention that jointly implemented Health Coaching and Service Navigation among a sample of adults ages 18–26 under supervision of the local probation department (*n* = 148). The study sought to increase participants’ healthcare utilization (i.e., recent doctor visit, health insurance coverage, having a usual source of care). Participants provided written informed consent to receive 12 weeks of health coaching and up to 6 months of weekly service navigation support. The intervention was delivered in-person; additional service navigation support was provided by phone or text messages if requested (Ojeda et al., 2021). At 6-month follow-up, a subsample of participants (*n* = 66) that were retained until 6-months reported statistically significant improvements in two indicators of healthcare access. Specifically, participants reporting having a doctor visit in the past 6 months increased from 36% at baseline to 71% at 6-months (*p* < 0.001), health insurance coverage increased from 64% at baseline to 88% at 6-months (*p* < 0.01) and the proportion of participants reporting a usual place of care rose from 55 to 70% at 6-months (*p* = 0.09) (Ojeda et al., 2024).

### Study contributions

While thousands of persons interact with the legal system annually, we could not identify any randomized clinical trials (RCTs) in the U.S. that specifically address JIA’s health literacy, highlighting a clear need to develop and test health literacy interventions for this underserved and under-researched community (Puglisi et al., 2017). Despite the increased use of Health Coaching in general populations, to our knowledge, it has not been integrated into healthcare access or health literacy interventions for JIA (Hill et al., 2015) nor has Health Coaching been paired with Service Navigation to support sustained use of healthcare among JIA. This protocol details the procedures for an RCT testing the effectiveness of a novel intervention pairing Health Coaching and Service Navigation to build health literacy with the goal of improving healthcare access among adult JIA ages 18 and older.

## Methods

### Conceptual model

We adapted the systems-level Gelberg-Andersen Behavioral Model of Service Utilization, which has been used to identify factors shaping healthcare use (Aday, 1993; Andersen, 1995; Stein et al., 2007). Key domains of this model include *Predisposing* (i.e., socio-demographics) and *Enabling* factors (e.g., marital status, housing, food security, transportation) which can support healthcare access and maintained use (Aday, 1993; Andersen, 1995; LeBel, 2012). Additional domains include *Need*-related factors (e.g., self-identified health, perceived need for care, clinically evaluated health) and *Health behaviors* including the use of health-promoting activities (e.g., diet, exercise, sleep) (Aday, 1993; Andersen, 1995). These constructs are measured in the baseline and follow-up surveys.

### Study aims & quantitative hypotheses

This study’s primary objective is to assess the effectiveness of a health literacy intervention (i.e., the University of California San Diego Re-Entry Community Linkages [UCSD RELINK] Program) for improving JIA’s healthcare access. Our specific aims for this longitudinal, mixed-methods randomized clinical trial (RCT) are to quantitatively assess the impact of the UCSD RELINK coach-guided health literacy intervention (i.e., health coaching and service navigation) vs. a control group (i.e., self-study of the health coaching program, and brief service navigation support) on healthcare utilization at 6-months and maintenance at 12-months. Secondary quantitative outcomes include assessing health insurance coverage and having a usual source of care. Using qualitative methods, we also seek to understand the impact and maintenance of the intervention at 6 and 12-months.

We hypothesize that participants receiving the coach-guided health literacy intervention will be more likely to report healthcare use at 6 months versus those assigned to the self-study control group. Similarly, we hypothesize that coach-guided health literacy intervention participants will be more likely to report having 1) health insurance and 2) a regular source of care at 6 month follow-up versus control group participants. Maintenance of these outcomes will be assessed quantitatively at 12 months. Additional exploratory outcomes include the types of health services used, type and continuity of health insurance coverage, reasons for being uninsured, delayed care seeking (i.e., has delayed getting medical care in the past 6 months), number of days in hospital, and satisfaction with care.

### Study setting

This study is based in San Diego, California, USA and it is home to > 3.5 million racially and ethnically diverse persons (City of San Diego, 2020). Currently, 35% of the county’s residents are Hispanic, 5.6% are African American, 43% are non-Hispanic White, and 13% are Asian (U.S. Census Bureau, 2023). Local statistics mirror national trends and underscore the disproportionate burden of incarceration on minorities (Carson, 2023). In 2022, local jails detained ~ 4,055 persons/day. During the COVID-19 pandemic, Hispanics and African Americans accounted for 57% of the jail population, but only 39% of the total population (SANDAG, 2023) Persons exiting prison and jail may be supervised by Probation or Parole. For example, in 2023, 42% of local probationers were Hispanic and 23% were African American (vs. 30% White) [total number of probationers > 20,000] (San Diego Probation Department, 2024).

### Ethics & consent

This study was approved by the University of California San Diego Human Subjects Research Protections Program (#161,903). All participants will provide informed consent to enroll in the study. No individual-level data are presented in this manuscript.

### Recruitment & venues

We will use venue-based recruitment to identify eligible individuals at community sites including three local Probation Department field offices, a resource center funded by the District Attorney’s Office, and local employment offices serving JIA.

### Sample & eligibility

This study will enroll 300 persons who meet the following eligibility criteria: is age 18 + , San Diego City resident (i.e., Central, South, Southeast regions), not incarcerated at enrollment, justice-impacted within the last 3 years (i.e., under supervision of probation or parole or court-diverted), available for the duration of the study period, has cell phone access, can provide informed consent, and has conversational English skills. Individuals who participated in the pilot study are ineligible due to prior receipt of the intervention; see ‘Pilot Study’ section, above for details (Ojeda et al., 2021). Adults who have current or prior convictions related to sexual violence/abuse (California Penal Code [PC] 290), lewd acts with a child (PC 288), child abduction (PC 280), or arson (PC 451) are also ineligible due to complications resulting from required interactions with the court and law enforcement agencies that oversee their supervision.

### Orientation & informed consent

Eligible JIA will complete a 90-min orientation led by the study’s recruiter, who will describe the study, answer questions, and obtain informed consent. Participants will complete a locator form and will be randomized to the control or treatment group, described below.

### Intervention

The UCSD RELINK intervention involves a Health Coaching psychoeducational program and Service Navigation support. The Health Coaching curriculum will be available in a printed workbook and on the study website which will host a digital workbook as well as videos that offer participants an audio-visual version of the workbook to facilitate learning for low literacy audiences. Table 1 summarizes the Health Coaching modules which are founded on cognitive-behavioral theories suggesting that emotions and behaviors influence our evaluation of the social and physical contexts in which we live (Beck & Beck, 1995). Individuals who have negative beliefs or skills deficits, such as poor emotional regulation, poor problem solving/self-advocacy skills, may have negative emotions and potentially, engage in health-damaging behaviors. Alternatively, persons with greater social and cognitive skills, such as goal setting, self-insight, executive functioning, may exhibit greater engagement in health-supporting behaviors. The Health Coaching program is designed to support participants’ acquisition self-insight and promote self-efficacy across a range of health and healthcare utilization domains (Bath, 2008; Berghuis, 2018; Brun & Rapp, 2001; Epstein & Street, 2011; Levenson & Willis, 2019; Miller & Rollnick, 2012; Wachtel & McCold, 2001). Health Coaching module-completion certificates and a final diploma are provided to reinforce learning and goal attainment.

**Table 1:** UCSD RELINK Health literacy curriculum to address disparities in healthcare access. (Available via a printed workbook and electronically via the study website as an electronic document or videos)

Module type	Module titles	Module learning objectives
**Core modules** *All required*	1. Program Orientation 2. Vision & Values 3. Goal Setting & Problem Solving 4. Stimulus Control 5. Navigating Health Services & Insurance 6. Communicate Effectively with Providers 7. Preventive Care (vaccines) 8. Using Technology to Support Your Health 9. Wrap Up	1. Build rapport with your Health Coach 2. Identify your core values 3. Set & achieve goals by creating manageable steps 4. Shape your physical & social environment to support your goals 5. Understand & use the healthcare system to support your health 6. Learn to advocate for yourself in healthcare visits 7. Learn about vaccines to protect your health 8. Learn to use technology to access health & social services 9. Review what you have learned!
**Emotional health** *Select 1*	1. Mindfulness & Stress Reduction 2. Mental Health 3. Loss & Grief	Learn to be deliberate in your life by developing positive strategies to manage emotions, stress, anxiety, & grief
**Physical health** *Select 1*	1. Physical Activity 2. Healthy Eating 3. Sleep Hygiene 4. Substance Use	Learn to nurture strong physical health throughout your life by developing healthy habits
**Healthy relationships** *Select 1*	1. Effective Communication & Empathic Listening 2. Conflict Resolution & Compromise 3. Boundaries in Relationships	Learn to build stable & supportive relationships through communication skills, boundary setting, and conflict resolution
**Life skills** *Unlimited selection*	1. Financial Literacy 2. Educational Roadmap 3. Employment Roadmap 4. Parenting Resources	Learn to support your life goals by planning your finances, strengthening your education, and career and using local resources to enhance your family’s wellbeing

Service Navigators will provide participants with resource identification, referrals and linkages, and they may need to provide mentorship with life skills. For example, an uninsured participant may be connected to a public health insurance application navigator in order to obtain health insurance, participants may be assisted in identifying a health care provider that is convenient to their work or home or participants may receive help in scheduling appointments with providers. Table 2 describes the life skills topics addressed in the Service Navigation workbook which will be available in a printed and digital format on the study website.

**Table 2:** UCSD RELINK service navigation resources and materials. (Available via a printed workbook and electronically via the study website as a document)

DOMAIN	TOPIC COVERED	DOMAIN	TOPIC COVERED
**Introductory Materials**	• UCSD RELINK CARE Checklist • Using the UCSD RELINK Website • UCSD RELINK Skills for Success Menu	**Health Literacy Handouts**	• Insurance: Understanding & Using Health Insurance • Insurance: Understanding Medicare • Insurance: Medi-Cal • Insurance: Medi-Cal Transportation Benefits • Insurance: Difference Between SSI and Medicare • Healthcare: Doctor Visit Preparation Checklist • Healthcare: Family Medical History • Healthcare: Preventative Care Checklists for Women • Healthcare: Preventative Care Checklists for Men • Healthcare: Vaccine Information and Checklist • Mental Health: Inclusive Mental Health Resources • Mental Health: Constructive Worry Sheet
**Financial Literacy Resources**	• Useful Tips & Resources • Budget Worksheet		
**Digital Literacy Resources**	• Connecting Through Email • Engaging with Social Media • Seeing Providers Through Tele-Health		
**Education Resources**	• Roadmap & Resources		
**Employment Resources**	• Roadmap & Resources • Resume Builder Template • Transportation: MTS Reduced Fares & Passes • Getting a Driver’s License	**Relationship Health Resources**	• Healthy Relationships
		**Substance Use Resources**	• Understanding Fentanyl
**Housing Handouts**	• Apartment Rental Checklist & Resources		
		**Goal Setting Resources**	• Time Management & Goal Setting for Success • Monthly Planner • Weekly Calendar Template
**Legal Support Resources**	• Understanding Your Probation		

### Interventionist credentials & training

For this study, the interventionists will include Health Coaches and Service Navigators. *Health Coaches* will have graduate-level training as licensed therapists (e.g., Marriage & Family Therapist), prior experience serving JIA and deep knowledge about the region. *Service Navigators* will be community members with lived experience or history of serving JIA by providing community resources per participants’ needs (e.g., gender, race/ethnicity, faith, geographic region, income level). Navigators may have university-based education (e.g., Associate of Arts, Bachelor of Arts, Master of Public Health, etc.) in fields adjacent to social services or health professions, but this is not required. Service Navigators will have established relationships with local institutions that explicitly serve the study’s target population.

Health Coaches will undergo standardized training of the intervention, including studying written training manuals, the Health Coaching curriculum workbook, and training videos that describe the intervention philosophy, components and staff roles. Health Coaches will also meet with the principal investigator (PI), project manager, data analyst, and lead mental health faculty advisor to complete the training. The Health Coaches will have prior training in the use of patient-centered, team-based, trauma-informed, and strengths-based care, Mental Health First Aid, de-escalation techniques, SMART Goal Setting, and Case Management (Bath, 2008; Berghuis, 2018; Bjerke & Renger, 2017; Brun & Rapp, 2001; Conzemius & O'Neill, 2009; Epstein & Street, 2011; Gelkopf et al., 2016; Hadlaczky et al., 2014; King, 2017; Levenson & Willis, 2019; Rapp, 1998; Richmond et al., 2012; SAMHSA, 2014a, 2014b; Wallace et al., 2011; Woods et al., 2013). Service Navigators will be trained in the study’s resource directory and key partners and best practices in survey administration and how to administer the online baseline intake survey. Service Navigators and Health Coaches will be trained in documenting services provided, incentives, and adverse events using the study’s case management system. The Health Coach and Navigator will receive ongoing supervision and annual refresher training. Administrative staff (i.e., PI, project manager, data analyst) will meet weekly with the staff to debrief.

All study personnel will also complete university-approved human subjects research protections programs certifications (i.e., Social and Behavioral Research; Biomedical Responsible Conduct of Research) (The CITI Program, 2017) and online trainings focused on effective team-work and remote-work to support participant- and study-staff-facing activities.

### *Study arms & randomization*

After being screened for eligibility, participants will be randomized 1:1 to the treatment or control groups, which are described below and summarized in Table 3. A stratified permuted block randomization will be applied to assess the effect of a health literacy intervention (UCSD RELINK) on recent healthcare use among JIA, comparing a coach-guided treatment group versus a self-study control group. A random permutation method with block sizes 4, 6, and 8 will be used where within each block, half the participants will be randomly assigned to the treatment group and the remaining half will be assigned to the control group. We will use data for biological sex at birth to determine which strata an individual will be assigned to (i.e., male or female) before randomizing the participant to an intervention group. Given the small proportion of female JIA, this approach ensures the treatment and control groups will be balanced within biological sex. The randomization list will be generated using Stata v16 (StataCorp., 2019).

**Table 3:** Comparison of the UCSD RELINK intervention delivery by study arm

	**TREATMENT GROUP** **Coach-Guided**	**CONTROL GROUP** **Self-Study**
**Program Orientation**	** + **	** + **
**Health Literacy Intervention Access**		
Curriculum Workbook	** + **	** + **
Study Website	** + **	** + **
**Health Coaching**		
Introduction to Health Coach	** + **	**–**
Weekly Health Literacy Intervention Meetings (~ 45 min; 12 sessions over 6 months)	** + **	**–**
Complete 12 modules (9 Core, 3 Elective)	** + **	** + **
Intervention Delivery Mode	Real-time sessions	Self-Study
Completion Certificates & Final Diploma	** + **	** + **
**Service Navigation**		
Introduction to Service Navigator	** + **	** + **
Weekly Case Management Meetings (30 min; 12 sessions over 6 months)	** + **	**–**
Review of Service Navigation Plan	** + **	** + **
Intervention Delivery Mode	Real-time sessions; Emailed and written referral list	Emailed and written referral list
**Weekly study participation reminders**	** + **	** + **

If this item is provided, it is represented as: “ + ”

If this item not provided, it is represented as: “—”

### Power & sample size

A total of 300 individuals will be enrolled into the study. The power calculation was based on the primary outcome of recent healthcare utilization over the study period (yes/no) and analyzed with a mixed-effects logistic regression. Power was conservatively calculated for recent healthcare utilization at baseline, 6 and 12-months based on the assumption that 60% of the treatment group and 40% of the control group would report recent healthcare use (Ojeda et al., 2021). Accounting for an intraclass correlation coefficient of 0.4 and considering 30% attrition (of the 300 enrolled at baseline), we anticipate we will need 208 participants (104/group) to detect an odds ratio of 1.8 of recent healthcare utilization between the intervention and control group with at least 80% power and 0.05 two-tailed alpha.

### Treatment arm: coach-guided intervention

Participants randomized to the treatment arm will work with their assigned Health Coach and Service Navigator; sessions are scheduled on days/times that are convenient to staff and participants. Participants will engage with the 12-module health literacy curriculum during weekly sessions with their Health Coach, using the curriculum workbook or program website, per their preference, to facilitate learning and exercise completion. Health Coaches will meet with participants during 12 sessions lasting ~ 45 min throughout a 6-month period. The Health Coach will tailor the curriculum per their needs and interests and guide each participant through the reflection exercises. The curriculum is designed for in-person delivery but can also be implemented by video or phone sessions to ensure continuity of the intervention.

Participants will also receive up to 12 sessions of Service Navigation support, per participant preference, lasting 30-min over a 6-month period. At baseline, the Navigator will conduct the intake survey in order to develop the service navigation plan which identifies resources for participants’ most pressing immediate needs. Navigators and Health Coaches will assist with linkage to resources, goal-setting, and mentoring participants through role-playing and modeling of behaviors that support healthcare access and encourage participants to practice new communication and preventive-care skills in real-world settings (e.g., preparing for healthcare visits, self-advocacy). In partnership with the Health Coach and Service Navigator, participants will reflect on barriers and facilitators and develop personalized solutions to support healthcare service utilization. Participants will also receive a written and electronic emailed Service Navigation report including providers’ contact information for independent follow-up.

Importantly, the intervention adopts a team-based approach: the staff support participants through targeted monitoring meetings to support participant self-management, successes, to anticipate new needs, or exchange resources that may impact their shared work. This approach is influenced by the Collaborative Care Model (CCM); for example, CCM has been successfully used with reentrants for care coordination and service linkages across cross-disciplinary teams (Kim et al., 2019).

### Control arm: self-study group

Participants assigned to the control arm will work with their assigned Service Navigator for two sessions. During the first session, participants will receive the Health Coaching and Service Navigation workbooks, complete the baseline intake service navigation survey, and will be instructed that they will independently engage with the curriculum using the workbook or the study website (via a personalized log-in which tracks module completion). During the first session, the Service Navigator will assist the participant to establish their study website log-in credentials and how to access the Health Coaching modules on the study website. Participants who prefer to complete the printed curriculum will be instructed to submit photographs of the completed workbook pages to the study email to receive certificates of completion; these may be shared with their supervising officer or the court to demonstrate participation in activities that can benefit the participant reenter the community. During the second study visit, participants will receive a printed and digital (emailed) service navigation report which identifies recommended service providers and resources, including contact information so the participant can access these resources independently. Participants will have opportunities to ask clarifying questions at this visit and no further contact with the Service Navigator will occur.

### Baseline service navigation intake survey

Service Navigators will be trained to administer the baseline survey (~ 60 min) which is foundational to developing the individualized Service Navigation plan. Surveys will be accessed via password-protected computers which connect to the HIPAA-compliant Qualtrics survey software (Qualtrics, 2005). Participants will be interviewed in private spaces to protect confidentiality and receive a $20 gift card at the first visit.

### Dependent variables

Our primary outcome, recent use of health services is a dichotomous measure defined as any healthcare use in the past 6 months (vs. never received services or received services more than 6 months ago) (CDC/NCHS, 2021). Our secondary outcomes include health insurance status (defined as: uninsured vs. any insurance), type, continuity, and reasons for discontinuity or uninsured status, having a regular source of care (yes vs. no), care seeking (i.e., has delayed getting medical care in the past 6 months), and number of days in hospital (CDC/NCHS,^2021^).

### Covariates

Covariates mirror the concepts identified in the conceptual model. Predisposing variables include demographics factors (age, race/ethnicity, gender, sexual orientation, marital status, duration of residence in San Diego County, veteran and foster care status) (CDC/NCHS, 2021). Enabling items are education, employment, income, food security, housing status, informational and emotional social support, and transportation access (CDC/NCHS, 2021; Hahn et al., 2010; Hahn et al., 2014; Salsman et al., 2019; University of Michigan, 2007; USDA, 2012; Veterans Affairs, 2016; Williams et al., 2008). Need variables include self-rated health, and provider assessed health status (Cella et al., 2019; Center for Behavioral Health Statistics & Quality, 2019; UCLA Center for Health Policy Research, 2019). Mental health status is assessed using the Patient Health Questionnaire (PHQ-9), Generalized Anxiety Disorder Scale (GAD-7), and a Traumatic Event Screener (Elhai et al., 2008; Kroenke et al., 2001; Spitzer et al., 2006). Health Behavior items include sleep, exercise, diet, and substance use (CDC/NCHS, 2021; Center for Behavioral Health Statistics & Quality, 2019; Santiago et al., 2017).

### Follow-up surveys & incentives to support retention

We will provide escalating incentive gift cards for survey-based data collection visits conducted at 6-months ($30) and 12-months ($50); these data will enable the researchers to assess participants’ use of health-supporting institutions over time. At baseline, we will collect participant locator forms for up to 5 persons who can help contact the participant, if needed and send visit reminders via text/email/phone call, per participant preference. We will provide $5 participant gift cards for monthly check-ins over months 7–12 in order to promote participant retention.

### Statistical analyses for survey data

A separate password-protected document with participants’ names and study ID number managed by the database administrator will enable linkage of all surveys. Statistical analyses will incorporate the intent-to-treat (ITT) principle. Participants who are randomized will be included in the ITT population. A mixed-effects logistic regression (Liu, 2015) will be used to analyze the effect of the coach-guided intervention on the primary outcome, recent healthcare utilization (yes/no) over the study period. This approach captures the between-individual and within-individual variability due to the repeated measures. Time invariant covariates include gender and age at baseline in addition to other predisposing, enabling, and health behavior variables determined to be unbalanced at baseline (univariate *p* < 0.10) and associated with the outcome (univariate *p* < 0.15). Time-varying covariates include follow-up time, treatment assignment (intervention vs. control), and time-by-treatment interaction terms. The maximum likelihood estimation approach used in the mixed-effects model enables us to incorporate all participant data even among those with only baseline measures. Analyses will be conducted using Stata v16 (StataCorp., 2019). Methods analogous to the analysis of the primary outcome will be applied to secondary outcome measures: 1) health insurance and 2) a regular source of care. Bonferroni’s correction will be applied for multiple testing of secondary outcomes, so statistical significance will be compared to a *p* < 0.025.

### Analysis of key secondary outcome (safety)

All participants will be included in a safety analysis. Adverse Events (AEs) and Serious AEs (SAEs) will be listed by participant. AEs will be summarized by method of collection type, frequency, severity, relationship to study intervention, any change in study intervention, and number of participants per treatment. The frequency of AEs and SAEs will be described by group and compared using Fisher’s exact test.

### Missing data

Data will be routinely monitored by the investigators to ensure completeness; it will be visually inspected for errors, omissions, and data outside the limit ranges. A Consolidated Standards of Reporting Trials (CONSORT) diagram will be produced at the end of the study (Schulz et al., 2010). We expect that study loss to follow-up will be moderate (30%) due to our multi-pronged retention approach. If participants are reincarcerated, they will continue to be eligible to participate once they return to the community if they are within the 12-month study participation period. Per our retention plan, substantial efforts will be made to ensure complete follow-up. Rates of missing data and loss to follow-up will be reported. For the primary aim, the mixed-effects regression will yield consistent regression parameter estimates provided that the missing data are missing at random, a reasonable assumption when there are sufficient covariates considered in the analysis (Liu, 2015). Alternative missing data strategies, such as missing data imputation strategies or propensity weighting, will be considered based on the degree of missingness in the data but will not be applied without extensive sensitivity analysis. Under these various missing data strategies, the statistical analysis will be run and compared for consistency. When serious deviations are observed, the impact of the missing data will be evaluated to see if that is the source of the discrepancy.

### Qualitative interviews: sampling, data collection & analysis

This mixed-method RCT also involves individual, semi-structured qualitative interviews, which are expected to last ~ 30 min and will occur with a sub-sample of participants at 6 and 12-months (with escalating incentives of $30 and $50). Interviews will explore immediate satisfaction with the curriculum, impacts of the intervention and maintenance of the knowledge and skills learned. We expect that many participants will experience challenges to engaging with the healthcare system, though this may not apply to all participants. Thus, we will employ purposive sampling to identify participants (*n* = 40, stratified by arm) who reported ≥ 1 healthcare access barriers at baseline (e.g., no recent doctor visit, uninsured status, and/or no regular source of care) (Johnson, 1990; Patton & Patton, 2002). This approach will allow us to more deeply explore the impact of the intervention on the treatment group as well as how the control group engaged with the study materials and their perceived impact on the outcome of interest. We will also attempt to recruit participants who did not complete the 6-month follow-up survey to ensure that their perspectives are included. The minimum number of interviews conducted for qualitative studies is expected to range from 15–20 (Malterud et al., 2016). By this standard, we anticipate that our sample size of 20 participants per intervention arm (*n* = 40 total) will be adequate for achieving thematic saturation within key topics of interest.

We use a deductive thematic approach to analyze the qualitative interview data as described below. Interviews will be digitally recorded, transcribed, and analyzed using team-based consensus methods (Willms et al., 1990). Specifically, analysts will first develop primarily deductive codes by reviewing key questions from interview guides and 2–3 interview transcripts. They will independently test preliminary codes by applying them to a second set of 2–3 transcripts and then meet to assess consistency in code application (i.e., reliability), resolve discrepancies, enhance code definitions, and finalize the codebook. A template approach (Crabtree & Miller, 1992) will be used to support thematic analysis and publication development; we will compare participants’ responses to the topics of interest as well as emergent topics related to health care utilization and intervention impacts.

### Limitations

Potential challenges to study implementation include identification of eligible JIA and participant attrition. However, the study employs a venue-based recruitment strategy wherein organizations that serve justice impacted persons are targeted for outreach and recruitment. We employ escalating incentives, locator forms, and monthly check-ins during the follow-up period to ensure that we have the latest contact information. These strategies are designed to foster participant retention.

## Discussion

Studies to build health literacy among justice-impacted adults are sorely needed given the challenges this vulnerable community faces in coordinating across multiple systems to meet basic needs and improve or maintain health and wellbeing. This study will leverage the strengths of quantitative and qualitative longitudinal methods to provide a breadth and depth of understanding regarding the effectiveness of the health literacy intervention described in this study. If successful, results may shift the paradigm of service delivery for JIA from a focus on single issues to comprehensive strategies that address JIA’s knowledge, skills, and self-efficacy to support sustained healthcare access. Importantly, the knowledge and skills learned are also applicable across other life domains and effective strategies are urgently needed amidst an evolving social, economic, and healthcare landscape. The long-term goal of this work is to improve healthcare access and reduce health disparities among JIA. If proven effective, the UCSD RELINK health literacy intervention may inform theory for interventions designed to reduce health disparities among JIA, the content and approach of service delivery models for JIA, and policies to fortify JIA’s health in institutional and community settings. Findings will also significantly expand our understanding of best practices for engaging and retaining JIA in health and longitudinal studies. Results may also inform our understanding of the scalability of this intervention in clinical and non-clinical settings.

## Data Availability

Not applicable; no data are presented in this article describing the study protocol.

## Abbreviations

JIA: Justice-impacted adults
RCT: Randomized Clinical Trial
UCSD RELINK Program: University of California San Diego Re-Entry Community Linkages Program
